# An Atypical Presentation of Chronic Inflammatory Myelin Degeneration in Neuromyelitis Optica (NMO)

**DOI:** 10.7759/cureus.41803

**Published:** 2023-07-13

**Authors:** Sai Vishnu Vardhan Allu, Harsh R Parikh, Patrik Schmidt, Gabriel Alonso, Sneha Khanal, Ked Fortuzi, Misbahuddin Khaja

**Affiliations:** 1 Internal Medicine, BronxCare Health System, Bronx, USA; 2 Internal Medicine and Pulmonary Critical Care, Icahn School of Medicine at Mount Sinai/BronxCare Health System, Bronx, USA

**Keywords:** neuroinflammation, autoimmune encephalitis, autoimmune, neuromyelitis optica spectrum disorder (nmosd), plasma exchange therapy

## Abstract

Neuromyelitis optica (NMO) is an autoimmune disorder characterized by aquaporin-4 (AQP4) IgG autoantibodies. These autoantibodies induce chronic neuroinflammatory damage to the spinal cord and optic nerve. NMO clinically manifests as relapsing and overlapping neurodegenerative episodes of optic neuritis (ON) and transverse myelitis (TM). Contrasting from other autoimmune neurodegenerative disorders, NMO has a poor prognostic profile often involving permanent neurological disability. We present a case of a 65-year-old male who presented with a progressive weakening in his left upper and lower extremities with reduced sensation and was found to have an acute flare of NMO. We explore the broad symptomatology involved in the disorder along with relevant crucial imaging findings pointing toward the diagnosis of NMO. Finally, we discuss treatment modalities in the context of our patient’s clinical course and prognostic factors. Early intervention and suppression of relapse in this neuroinflammatory neurodegenerative disorder can help decrease the duration of acute flares and improve long-term outcomes for patients affected by NMO.

## Introduction

Neuromyelitis optica (NMO), formerly known as Devic’s syndrome, is a chronic autoimmune disorder characterized by anti-aquaporin-4 (AQP4) IgG antibodies that induce neuroinflammatory damage within the central nervous system (CNS) [1-3]. The IgG antibody binds to the AQP4 channels, triggering the classical complement cascade and granulocytes, lymphocytes, and eosinophil infiltration, initially targeting astrocytes and then oligodendrocytes leading to consequent demyelination and neurodegeneration of the optic nerve and cervical spinal cord [4-6].

Clinically, NMO manifests as relapsing patterns of optic neuritis (ON) and transverse myelitis (TM) with lesions of the cerebral cortex and brain stem [1-3]. Clinical symptoms for NMO-TM include diffuse patterns of neuromotor and sensory deficits in the extremities with superimposed neuropathic pain [1,2,4-6]. The sensorimotor deficits are variable in NMO-TM, ranging from mild paresthesia to full paresis [1-3]. Additionally, NMO-TM can present with bladder, bowel, and erectile dysfunctions [1,2]. NMO-related ON symptoms involve the onset of hazy vision with decreased high-contrast visual acuity on the Snellen vision test, deteriorating low-contrast visual acuity in color vision, and scotoma [2,6]. Uncommonly, NMO can also present with signs of cerebral neurodegeneration: nausea and vomiting, oculomotor disturbances, facial numbness, hypothalamic syndromes (narcolepsy, syndrome of inappropriate antidiuretic hormone secretion (SIADH), etc.), and thalamic pain syndromes [1-3,6].

In this report, we evaluate an atypical case of NMO with chronic relapsing neurological sensorimotor deficits and diffused unilateral hyperalgesia, but with no involvement of the optic nerve.

## Case presentation

This is the case of a 65-year-old Latin-American male, HF, with a past medical history of gout, chronic kidney disease (CKD), type 2 diabetes mellitus (T2DM), cerebral vascular accident (CVA) in 2018, hypertension, and coronary artery disease (CAD) managed with a coronary artery bypass graft (CABG) in 2016. HF presented to the emergency department with complaints of a four-day worsening weakness and paresthesia in the left upper and left lower extremities with decreased sensation. HF also reported complaints of a parietal headache with intermittent muscle spasms in the back and neck.

HF currently resides in a nursing home and has a 10-15 year smoking history and a history of cocaine abuse with last usage reported within the past year. HF had been hospitalized two times within the past three months with similar complaints of worsening left-sided weakness, neck spasms with headaches, and diffuse nonspecific left-sided hyperalgesia. Previous CT and CT scans of the head, neck, and spine had all been negative with no indications of stroke or structural lesions. A previous MRI of the brain was negative for signs of CVA but identified scattered periventricular white matter T2 intermediate foci, consistent with either small vessel ischemia or demyelinating white matter disease. Previously performed carotid duplex ultrasound revealed >50% stenosis of the right carotid artery and >75% stenosis of the left carotid artery. However, no vascular intervention was performed.

On presentation to the emergency department, the patient was vitally stable with both left upper and lower extremity weakness graded as 0/5. A non-contrast CT image revealed no indications for new-onset CVA. The only pertinent laboratory finding at this time involved an elevated white blood cell (WBC) count with a neutrophilic predominance. The patient was then admitted to the critical care unit (CCU) for an MRI of the cervical spine and further investigation.

**Figure 1:**
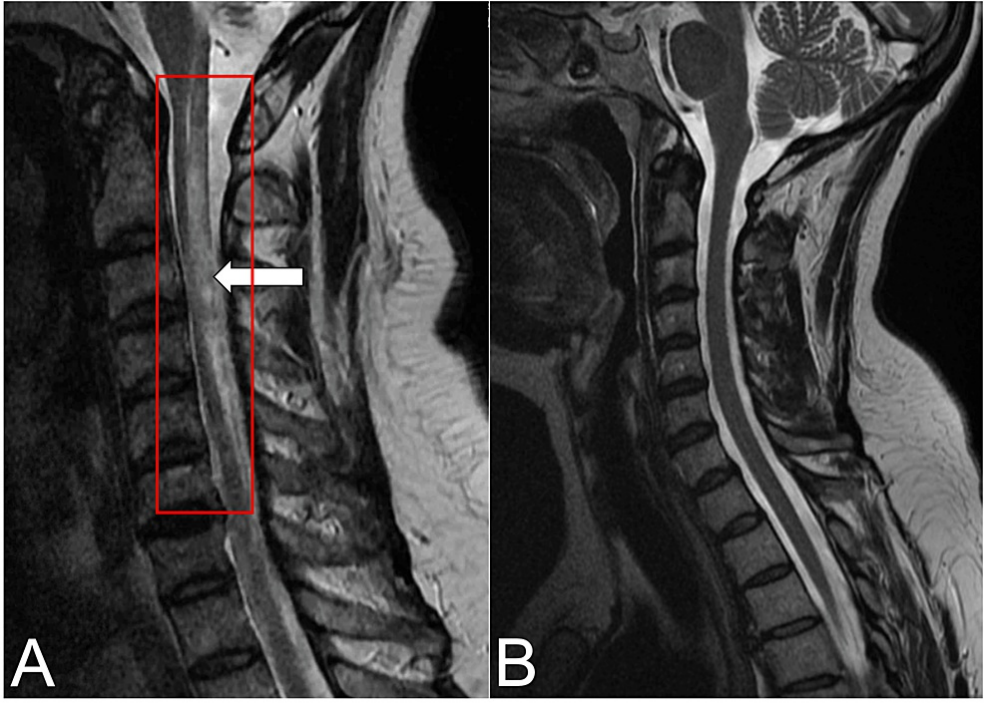
Sagittal view C-spine MRI Sagittal view T2-weighted C-spine MRI of patient HF demonstrates a diffuse increased signal intensity throughout the cervical spine cord (red box), representing significant cord edema, myelomalacia, and potential cord contusion (white arrow) (A). For comparison, a normal T2-weighted sagittal view C-spine MRI is provided from a separate 55-year-old patient (B) [7,8]. MRI: magnetic resonance imaging, C-spine: cervical spine

Upon arrival at the CCU, the patient was found to be experiencing new-onset right-sided weakness and decreased sensation in both the upper and lower extremities, graded 2/5. T2-weighted MRI of the cervical spine identified increased signal intensity throughout, demonstrating cord edema and swelling (Figure 1A) [7,8]. With no evidence of traumatic etiology, clinical investigations were directed toward a differential diagnosis of demyelinating neuroinflammatory disorders, multiple sclerosis (MS), neuromyelitis optica (NMO), myelin oligodendrocyte glycoprotein antibody disease (MOGAD), transverse myelitis, and acute disseminated encephalomyelitis (ADEM) [2-4]. On day 10 of admission, HF tested positive for anti-aquaporin-4 (AQP4) IgG antibodies, confirming a diagnosis of neuromyelitis optica without the involvement of the optic nerve [2,7]. With a confirmed diagnosis of NMO, initial clinical management consisted of a three-day course of dexamethasone and five sessions of plasmapheresis over 10 days, with long-term management with rituximab every six months. Of note, the patient’s hospital course was complicated by a non-ST-elevation myocardial infarction (NSTEMI) with secondary decompensating heart failure. The patient was intubated for five days and managed with dual antiplatelet therapy (DAPT) and furosemide.

In summary, HF experienced a 41-day hospital course. The patient received all five sessions of plasmapheresis during the inpatient stay and the first dose of rituximab for long-term clinical management. Post-intervention, HF demonstrated improving motor and sensory function in both the right and left extremities. Left upper and lower extremity strength improved from 0/5 to 2/5, and right upper and lower extremity strength improved from 2/5 to 3/5. HF was scheduled for outpatient physical therapy to continue strength improvement, outpatient three-month MRI to assess for change in cord swelling and edema, and monthly outpatient neurological clinic visits to assess long-term treatment efficacy.

## Discussion

Neuromyelitis optica (NMO) is a rare autoimmune condition that typically targets the spinal cord and optic nerves in the central nervous system. It may result in blindness, limb weakness or paralysis, excruciating spasms, loss of sensation, vomiting, hiccups, and bladder and bowel dysfunction [1-3]. Clinical features such as optic neuritis, myelitis, and brain stem syndromes are also commonly seen. Although there is no cure, there are several medical therapies available that can help control the illness and lower the likelihood of relapses [9]. The epidemiology of neuromyelitis optica (NMO) shows varying prevalence rates among different racial groups worldwide, with East Asians and Blacks having higher prevalence than Whites [9]. Autoimmune processes play a role in the neuromyelitis optica (NMO) etiology. Multifocal inflammation of the spinal cord and optic nerves is the hallmark of NMO, which also affects other parts of the central nervous system. Autoantibodies, specifically those directed against aquaporin-4 (AQP4) water channels, are thought to be the mediating factor [10]. These autoantibodies cause inflammation, demyelination, and axonal damage while rupturing the blood-brain barrier and activating the complement. At this time, it is unclear what exactly triggers the immunological response in NMO [11].

Numerous similar characteristics define neuromyelitis optica (NMO). These include myelitis, which results in extremity weakness, sensory loss, and paralysis, painful spasms, and optic neuritis, which causes eye pain and visual loss. Other symptoms involve nonspecific complaints involving the genitourinary and gastrointestinal tracts, excessive nausea and vomiting, seizures, and relapses [12]. In the case of our patient, the initial presenting symptoms were frequent headaches with left-sided hyperalgesia, intermittent muscle spasms of the neck, perirectal pain, and reduced extremity strength and sensation. These symptoms were progressive with minimal improvement in symptoms in between hospitalizations. His initial set of symptoms was broad; however, his MRI study did indicate a possible underlying demyelinating disorder.

Neuromyelitis optica (NMO) is a complex progressive neurological disorder that is difficult to diagnose. Along with a thorough review of the patient’s medical history and an assessment of their symptoms, a thorough physical and neurological examination is carried out [13]. Blood tests are used to find autoantibodies linked to NMO, such as anti-AQP4 and anti-myelin oligodendrocyte glycoprotein (MOG) antibodies [13]. CT scan and MRI of the optic nerve and spinal cord can also be helpful in working up the disorder and associated differential diagnoses [13,14]. Due to similar symptoms, differential diagnosis of illnesses such as multiple sclerosis is essential [14]. Differential diagnoses for neuromyelitis optica (NMO) include multiple sclerosis (MS), acute disseminated encephalomyelitis (ADEM), optic neuritis (ON), and transverse myelitis (TM) [15]. These conditions share overlapping clinical features with NMO, making accurate differentiation important for appropriate management and treatment. Furthermore, serologic studies in our patient also showed the presence of anti-AQP4 antibodies, confirming the diagnosis of NMO.

The goal of neuromyelitis optica (NMO) treatment is to abort the acute attack and prevent permanent harm. The standard treatment for acute NMO attacks is high-dose intravenous corticosteroids such as methylprednisolone. If corticosteroids are ineffective, plasma exchange (PLEX) may be employed. Future NMO attacks can be reduced by the administration of immunotherapies, which inhibit the immune system and reduce inflammation, especially in people who test positive for anti-aquaporin-4 (AQP4) antibodies [16]. Our patient received dexamethasone in the acute setting, followed by eventual plasmapheresis, and finally initiation of rituximab for long-term maintenance therapy.

The prognosis for neuromyelitis optica (NMO) varies. Studies have shown that the normal five-year mortality rate for NMO is between 22% and 30% [17]. NMO is a rare autoimmune disease that causes inflammation in the central nervous system and mostly affects the spinal cord and optic nerves [18]. As a result, the myelin sheath and supporting nerve fibers may be harmed or destroyed [19]. The prognosis for neuromyelitis optica (NMO) can be influenced by several factors; the severity and frequency of relapses, the extent of optic nerve and spinal cord damage, and the response to treatment all contribute to the prognosis [20]. Early diagnosis and prompt initiation of appropriate therapies may help in managing the disease and improving outcomes [2,3,9]. Individual variability in the course of the disease adds further complexity to prognosis assessment [3].

## Conclusions

Neuromyelitis optica is a progressive autoimmune neurological condition that targets the central nervous system, leading to a wide array of symptoms including muscle spasms and pain with paralysis, bladder and bowel dysfunction, loss of sensation, and even blindness. The underlying cause is believed to be multifocal inflammation in the spinal cord and optic nerves due to the presence of AQP4 antibodies. Prompt treatment with glucocorticoids during acute attacks, followed by plasmapheresis in severely symptomatic patients can help abort acute attacks of NMO. Long-term therapy with immunosuppressive agents, such as rituximab, as in our patient, can help prevent relapses. Early recognition and initiation of medical therapy can help manage acute flares of the disorder and improve clinical outcomes in this rare neurodegenerative disorder.
